# Advancing ecological risk assessment on genetically engineered breeding stacks with combined insect-resistance traits

**DOI:** 10.1007/s11248-019-00185-8

**Published:** 2020-01-17

**Authors:** Justin McDonald, Andrea Burns, Alan Raybould

**Affiliations:** 1 Product Safety, Syngenta Crop Protection, LLC, Research Triangle Park, NC USA; 2 Science, Technology and Innovation Studies and Global Academy of Agriculture and Food Security, University of Edinburgh, Edinburgh, UK

**Keywords:** Ecological risk assessment, Insecticidal proteins, Transgenic, Problem formulation, Breeding stacks

## Abstract

To inform the ecological risk assessment (ERA) of a transgenic crop with multiple insecticidal traits combined by conventional breeding (breeding stack), a comparative field study is customarily conducted to compare transgenic protein concentrations in a breeding stack to those in corresponding component single events used in the breeding process. This study tests the hypothesis that transgenic protein expression will not significantly increase due to stacking, such that existing margins of exposure erode to unacceptable levels. Corroboration of this hypothesis allows for the use of existing non-target organism (NTO) effects tests results, where doses were based on the estimated environmental concentrations determined for a component single event. Results from over 20 studies comparing expression profiles of insecticidal proteins produced by commercial events in various combinations of conventionally-bred stacks were examined to evaluate applying previously determined no-observed-effect concentrations (NOECs) to stack ERAs. This paper presents a large number of tests corroborating the hypothesis of no significant increase in insecticidal protein expression due to combination by conventional breeding, and much of the variation in protein expression is likely attributed to genetic and environmental factors. All transgenic protein concentrations were well within conservative margins between exposure and corresponding NOEC. This work supports the conclusion that protein expression data generated for single events and the conservative manner for setting NTO effects test concentrations allows for the transportability of existing NOECs to the ERA of conventionally-bred stacks, and that future tests of the stated hypothesis are no longer critically informative for ERA on breeding stacks.

## Introduction

Insect pests in agricultural fields can cause immense damage to crops, lowering yields and decreasing grower income. Growers have many options to decrease insect pest populations in their crops including the application of chemical and biological insecticides (e.g., *Bt* microbial pesticides), and the use of seeds with insect-resistance traits introduced through plant transformation. Transgenic crops that have been engineered to express genes encoding proteins that are toxic to specific insect pests have been labeled as plant incorporated protectants (PIP) by the United States Environmental Protection Agency (US EPA) (Matten et al. 2012). These PIPs provide benefits to growers as protection to optimize yield and benefits to the environment, as they may reduce the need for and localize the application of insecticides (Carpenter et al. 2002; Brookes and Barfoot 2017a, b). This environmental benefit, however, does not preclude transgenic crops with insecticidal traits from environmental and ecological risk assessment. Because these proteins have toxic activity to insect pests, it is important to evaluate the risk that these proteins may affect non-target organisms (NTOs) due to cultivation of transgenic crops in the environment (Carpenter et al. 2002; Romeis et al. 2008).

Ecological risk assessment is most effective when protection goals are clear and a case-by-case problem formulation is conducted to evaluate the overall risk hypothesis that no ecological harm will occur due to the cultivation of transgenic crops (Wolt et al. 2010; Raybould 2007). Problem formulation is the first step in any risk assessment. Through problem formulation, pathways by which valued and potentially susceptible species may be exposed to the insecticidal proteins can be identified and testable hypotheses crafted to guide scientific study of those pathways to harm (Wolt et al. 2010). This provides guidance for design of experiments needed to aid risk assessment of any new transgenic crop (Wolt et al. 2010).

Many insecticidal traits have been introduced into commercial maize hybrids through plant transformation since the first commercialization of such a product in 1996 (ISAAA 2017). Seed developers quickly realized that combining different insecticidal traits would be beneficial because crops could be encountering multiple insect pest species (Edgerton et al. 2012). The additional protection offered by the expression of multiple insecticidal traits with activity against different insect pests is an obvious benefit. Another benefit is minimizing the potential for resistance by having a plant produce multiple insecticidal proteins that have different modes of action against the same insect pest (Storer et al. 2012).

Once the trait genes have been introduced into individual lines through transformation, the combination of multiple desirable traits into one germplasm can be achieved through conventional breeding techniques. The breeding techniques are then no different for traits that are native to the plant introduced from a different cultivar. For genetically modified crops, this has been referred to as “trait stacking” and the products have various names such as “stacks,” “breeding stacks,” and “combined events products” (CLI 2011). The International Service for the Acquisition of Agri-Biotech Applications estimated that in 2017, 77.7 million hectares were planted with stacks globally (ISAAA 2017).

Stacks are regulated to a lesser degree overall; however, some agencies apply more scrutiny to the safety evaluation than others. Regulatory agencies customarily recognize that the characterization studies to assess the allergenic and toxic potential of the encoded protein performed initially for risk assessment of the single event applies to the same protein produced by a stack. This is transferable if that stack has been confirmed to express the same genetic elements received from the parent plant expressing that transgene as a single event. However, initially there may have been uncertainty over the potential for interactions to occur between transgene products from multiple single events. One concern related to this uncertainty is whether the production of a transgenic protein may increase due to the presence or production of another in the same plant (Raybould et al. 2012). A comparative testing strategy has been used to evaluate this concern, which is part of the customary framework for ecological risk assessment of breeding stacks in regards to insecticidal proteins (Raybould et al. 2012).

Regulatory agencies in multiple countries require that the abundance of the transgenic proteins produced by a stack be compared to that of the corresponding component single events. This type of experiment is required with differing conditions by regulatory agencies, including the United States Environmental Protection Agency (US EPA) and the European Food Safety Authority (EFSA). From an ecological risk assessment perspective, this experiment is meant to test the hypothesis that the concentrations of transgenic proteins are not significantly increased in the stack when compared to the component single events such that existing margins of exposure (MoE) are eroded to unacceptable levels (Raybould et al. 2012). Corroboration of this hypothesis allows for the use of existing NTO effects test results, where test concentrations were set based on the Estimated Environmental Concentrations (EECs) determined for a component single event, to inform ERA of a breeding stack.

The use of EECs in risk assessment of transgenic crops is similar to that for pesticides. Typically, exposure is computed using measured concentrations and other factors such as body weight and daily consumption when appropriate. Toxicological experiments are conducted to determine the highest dose at which no harmful effect is observed, referred to as a no-observed-effect concentration (NOEC) or a no-observed-adverse-effect concentration (NOAEC) (Romeis et al. 2011). For the proteins that were the subject of this work, a single test concentration many times higher than the EEC was selected. If no effects are observed at this conservative level, then the NOEC is at least this single test concentration. The NOEC is divided by the exposure level to provide a quantitative value from which risk may be judged, referred to as a margin of exposure (Raybould et al. 2012). A MoE equal to or greater than one would indicate negligible risk from exposure in a field setting (Raybould et al. 2012).

The mean concentrations that underlie EEC calculations are computed from concentrations measured in plant samples collected from field grown plants at multiple locations. Such data are generated as part of a typical regulatory study conducted to establish the transgenic protein expression profile for any new genetically-engineered crop.

Using existing NTO effects test results for the risk assessment of stacks relies on robust estimation of the EEC for the single event, testing for harmful effects at concentrations several times greater than the single event EEC and no biologically relevant increases in insecticidal protein expression levels in the stack. We define a biologically relevant difference as one that leads to unacceptable MoEs: when the EEC is greater than the NOEC (Raybould et al. 2012; Romeis et al. 2008). Corroboration of the hypothesis that differences in insecticidal protein concentrations in stacks compared to the single component event are not biologically relevant would suggest that regulatory requirements to compare transgenic protein concentrations in stacks and component events could be reduced in some circumstances.

Comparison of transgenic protein concentrations in stacked plants to those in component single event plants is conducted by a designed experiment. Plants of each component single event and the stack are grown together in a field trial with replication, and tissue samples are collected and analyzed to quantify the abundance of the transgenic proteins. Statistical comparisons test the null hypothesis that protein concentrations in the stack are no different than those of the component single event. Over the years, regulatory agencies have requested that this experiment include additional parameters, including analysis of tissue types collected at multiple growth stages, and replication of the field experiment at multiple locations. Additionally, a high level of scrutiny has been applied by some regulatory authorities when comparisons of transgenic protein concentrations between a stack and the component single events result in statistical significance without considering relevance to risk assessment.

A statistically significant higher concentration of an insecticidal protein in a stack compared with a single event does not necessarily mean that new NTO effects tests are required. First, if many comparisons are made (i.e., the hypothesis of no difference is tested many times simultaneously) it is likely that some will be statistically significant by chance. Second, if the increased concentrations in the stack are relatively small, the NTO effects test results may still be useful. For example, in the case of Bt11 × MIR604 maize, a few statistically significant differences were observed in which the amount of the insecticidal proteins (Cry1Ab and mCry3A) were higher in the stack than in Bt11 or MIR604 maize (Raybould et al. 2012). The relative increase of the protein concentration measured in the stack and single was calculated and the MoE for potentially exposed NTOs was reduced proportionally to determine if the difference was biologically relevant (Raybould et al. 2012). In this case, the relative increase was no greater than 1.5 fold and did not overturn margins of exposure in any case (Raybould et al. 2012). Therefore, the risk of adverse effects to NTOs from exposure to those proteins produced by Bt11 × MIR604 maize was deemed negligible: unchanged from that for each of the component single event maize Bt11 and MIR604. New NTO effects tests should only be necessary to inform risk assessment if the concentration of an insecticidal protein is consistently greater than the component single event at a biologically significant level.

The objective of this work was to examine the results from multiple studies conducted to compare transgenic protein concentrations between several stacks and component single events, and then to examine how the observed statistically significant increases in expression levels relate to NOECs. We used data from 22 different protein expression studies with field trials in four different countries conducted on six different maize stacks. These maize stacks included various combinations of the transgene traits from plants derived from transformation events Bt11, MIR604, MIR162, 5307, and GA21 (Table 1).

This paper is divided into two sections to clearly communicate how the data were generated and the evaluations that were made to achieve the objective. First, the number of statistically significant increases of insecticidal protein concentrations in breeding stacks compared to component single events observed in all 22 studies were totaled. Then, the biological relevance of those significant increases was evaluated.

## Testing for significant difference: stack versus single events

### Materials and methods

Data on levels of the transgenic proteins were generated from individual field studies conducted in locations of commercial maize cultivation, which satisfied regulatory requirements for cultivation approvals in the USA, Canada, and Argentina, and import approvals from maize grain importing countries. Similar studies were conducted for the two stacks, Bt11 × MIR604 × GA21 and Bt11 × GA21, in which the field trials were located in two separate European field trial sites and one located in The Republic of South Africa (Table 1).

**Table 1:** Summary of comparative protein expression studies

Stack	Field trial locations (year)
3272 × Bt11 × MIR604 × TC1507 × 5307 × GA21	Argentina (2012–2013)
Bt11 × MIR162 × MIR604 × TC1507 × 5307 × GA21	Iowa (2009)
Bt11 × DAS-59122–7 × MIR604 × TC1507 × GA21	Iowa (2009); Iowa, Pennsylvania, Iowa (2012)
Bt11 × MIR604 × TC1507 × 5307 × GA21	Iowa (2009)
Bt11 × MIR162 × TC1507 × GA21	Hawaii (2008), Nebraska (2010)
3272 × Bt11 × MIR604 × GA21	Illinois (2007)
Bt11 × MIR162 × MIR604 × GA21	Illinois (2006)
Bt11 × TC1507 × GA21	Iowa, Wisconsin, Pennsylvania (2014)
3272 × Bt11 × GA21	Florida (2006)
Bt11 × MIR162 × GA21	Illinois (2006), Wisconsin, Minnesota, Pennsylvania (2012)
Bt11 × MIR604 × GA21	Illinois (2006), Romania (2008), Spain (2008)
Bt11 × MIR604	Illinois (2005)
Bt11 × GA21	Illinois (2005), Romania (2008), Spain (2008), RSA^a^ (2009)
MIR604 × GA21	Illinois (2005)

Each comparative field trial included stack and each of the corresponding component single events

^a^*RSA* Republic of South Africa

Each stack was produced by conventional breeding of various combinations of maize lines derived from the individual transformation events Bt11, MIR162, MIR604, 5307, TC1507, DAS-59122-7, and GA21 maize. Bt11 maize produces a truncated Cry1Ab insect-control protein, which has activity against certain lepidopteran pests (ILSI CERA 2011), and phosphinothricin acetyltransferase (PAT) protein, which confers tolerance to herbicide products containing glufosinate (Hérouet et al. 2005). MIR162 maize produces Vip3Aa20 protein, for control of certain lepidopteran pests (Lee et al. 2003), and phosphomannose isomerase (PMI) protein, which is a selectable marker enabling transformed plant cells to utilize mannose as a primary carbon source (Reed et al. 2001). MIR604 maize produces modified Cry3A protein (mCry3A), which has activity against certain coleopteran pests (Walters et al. 2008), and the PMI protein. 5307 maize produces eCry3.1Ab protein, which has activity against certain coleopteran pests (Oyediran et al. 2016), and the PMI protein. TC1507 maize produces Cry1F, insect-control protein, which has activity against certain lepidopteran pests (Baktavachalam et al. 2015), and the PAT protein. DAS-59122-7 maize produces Cry34Ab1 and Cry35 Ab1 proteins, which have activity against certain coleopteran pests (Baum et al. 2004), and the PAT protein. GA21 maize produces a double-mutated 5-enol pyruvylshikimate-3-phosphate synthase protein (mEPSPS), which confers tolerance to herbicide products containing glyphosate (Dill 2005). Specific stacks were selected for this data analysis (Table 1) to offer multiple instances of specific trait combinations that may reveal directional trends of expression (Table 2).

**Table 2:** Number of breeding stacks with distinct combinations of insecticidal proteins

Distinct insecticidal protein combination	No. of stacks with combination
Cry1Ab × mCry3A	8
Cry1Ab × Vip3Aa20	4
Cry1Ab × eCry3.1Ab	3
mCry3A × Vip3Aa20	2
mCry3A × eCry3.1Ab	3
Vip3Aa20 × eCry3.1Ab	1
Cry1Ab × mCry3A × Vip3Aa20	2
Cry1Ab × mCry3A × eCry3.1Ab	3
Cry1Ab × mCry3A × Vip3Aa20 × eCry3.1Ab	1

In accordance with the problem formulation regarding environmental and ecological risk assessment, only the data for insecticidal proteins were considered. Data for the insecticidal proteins Cry1Ab, eCry3.1Ab, mCry3A, Vip3Aa20 produced by Syngenta were included in this paper.^1^ The proteins PAT, PMI, and mEPSPS were not included since neither have insecticidal activity. Cry1Ab and mCry3A concentrations generated from the comparative protein expression studies for the stacks Bt11 × GA21, MIR604 × GA21, and 3272 × Bt11 × GA21 were also included in the analysis. These three stacks did not include a combination of two or more insecticidal traits. However, these were included in the analysis as many regulatory agencies require this test regardless of trait function (e.g., insecticidal, herbicide tolerance, etc.).

Each comparative protein expression study was conducted with a similar study design. Samples for protein expression analysis were collected from plants in five replicate plots for each of the stack and its component single events, all arranged in a randomized complete block design within each field trial. Maize plants of the stack and its corresponding component single events grown for each field trial were of the same genetic background. Multiple tissue types at multiple growth stages were collected and analyzed for each study. The types of plant tissues collected overall included leaves, roots, pollen, and kernels at various stages in development (Table 3). The field trials were maintained according to normal agricultural practices for the region, including the use of pesticides necessary to maintain plant health.

**Table 3:** Summary of maize plant tissue samples collected across studies

Tissue type	Growth stage^a^		Sample description
Leaves		Mid-Whorl, Late-Whorl, R1, R6	All healthy leaves from one plant
Roots		Mid-Whorl, Late-Whorl, R1, R6	All roots from one plant excluding above-ground brace roots
Pollen		R1	Pooled from multiple plants
Kernels		R6, senescence	All kernels from the primary ear of one plant

^a^Abendroth et al. (2011); Mid-Whorl includes a range of stages from V5 to V8. Late-Whorl includes a range of stages from V9 to V12

Each sample was put directly on dry ice immediately after removal from the maize plant and stored frozen. Frozen samples were ground using a commercial food processor with dry ice. Proteins were extracted by homogenization in a buffer validated for use on each protein and tissue type at the time each study was conducted. Extraction methods were optimized over the years to accommodate efficient use of a single extract for multiple proteins. Current validated extraction methods include the use of phosphate-buffered saline with 0.05% Tween 20 (pH approximately 7.4) for all four proteins (Cry1Ab, mCry3A, Vip3Aa20, and eCry3.1Ab) and tissues except for eCry3.1Ab in pollen for which a borate buffer^2^ (pH approximately 7.5) was used. Extracts were analyzed by an Enzyme-Linked Immunosorbent Assay (ELISA) specific for a target protein. The concentration of each protein was interpolated from a standard curve and then converted to microgram (µg) of protein per gram (g) of sample. Concentrations were available on the basis of both fresh weight and dry weight by way of a conversion using the moisture content percentage for each sample. Analysis of variance was used within each study to compare mean protein concentrations in the combined events product with the corresponding single event on a dry-weight basis for each tissue type and growth stage. In each analysis, the statistical significance was determined using a standard F-test at the customary alpha level of 0.05.

To examine the results over multiple studies, the amount of statistically significant increases of Cry1Ab, mCry3A, Vip3Aa20, and eCry3.1Ab concentrations in breeding stacks compared to component single events were totaled for each tissue type and across tissue types.

## Results

Over all four proteins, 50 statistically significant differences were observed out of 204 comparisons of concentrations between a stack maize with two or more insecticidal proteins and each corresponding component single event maize (Table 4). Approximately half of those differences (26 of 50) were due to higher concentrations in the stack compared to those of the corresponding component single event (Table 4). At the alpha level of 0.05 with 204 comparisons, 10 comparisons would be expected to show a significant difference due solely to random chance. A similar proportion of significant differences were observed for the 44 statistical comparisons of the breeding stacks Bt11 × GA21, GA21 × MIR604, and 3272 × Bt11 × GA21 for which only one PIP was involved.

**Table 4:** Frequency of statistical comparisons with results significantly different between stack and corresponding component single event

Protein	Tissue type	No. of tests	No. of significant differences (α = 0.05)	
			Stack ≠ single	Stack > single
Cry1Ab	Leaf	34	10	8
	Root	30	4	3
	Kernel	22	6	5
	Pollen	7	3	2
	All	93	23	18
mCry3A	Leaf	22	8	2
	Root	23	3	1
	Kernel	12	4	0
	Pollen	4	2	2
	All	61	17	5
Vip3Aa20	Leaf	12	0	0
	Root	9	1	1
	Kernel	7	0	0
	Pollen	6	1	1
	All	34	2	2
eCry3.1Ab	Leaf	6	4	0
	Root	6	2	1
	Kernel	4	2	0
	All	16	8	1
Total		204	50	26

## Evaluation of biological relevance

### Materials and methods

The ERAs on the single events Bt11, MIR604, MIR162, and 5307 included worst-case EECs calculated from the highest mean concentration of the transgenic protein from a single field trial location in a plant tissue type most relevant to valued species of interest (Raybould et al. 2007; Raybould and Vlachos 2011; US EPA 2010; Burns and Raybould 2014). Valued non-target organisms rarely consume a diet comprising 100% of a particular tissue of a crop. Therefore, the EECs can be further refined to represent more realistic scenarios conforming to specific pathways of potential exposure. Predatory insects, for example, may be exposed to protein from crop tissue consumed by their prey. Measurements of the concentrations of transgenic protein in prey feeding on transgenic plant tissues (e.g., Head et al. 2001; Dutton et al. 2002) can be used to set dilution factors for calculating an EEC from a mean tissue concentration.

Measured concentrations of each insecticidal protein in various maize tissues were compiled from all studies (Table 1). The mean concentrations on a fresh-weight basis were evaluated against the EECs determined for the insecticidal protein produced by each of the Bt11, MIR604, MIR162, and 5307 maize events, and the smallest NOEC among species was tested against appropriate tissues ([Bt11: US EPA 2010], [MIR604: Raybould et al. 2007], [MIR162: Raybould et al. 2012], [5307: Burns and Raybould 2014]). The EECs were taken directly as cited in those published papers, with the exception of Cry1Ab (Bt11 maize). A published work providing tissue-specific Cry1Ab EECs was not available for this analysis. Therefore, fresh-weight based Cry1Ab concentrations were sourced from an unpublished report submitted to the EPA to support the risk assessment of Bt11 maize (Privalle 2003). The highest mean concentration for each tissue type reported by Privalle 2003 was used to represent each tissue-specific EEC for the Cry1Ab protein. The report by Privalle was cited in the Biosafety Registration Document (BRAD) for Cry proteins published by the US EPA in 2010 (Privalle 2003: MRID# 45879803 [US EPA 2010]). The Cry1Ab concentrations reported in this 2010 BRAD were sourced from one of the selected comparative studies (Table 1) and therefore, are not suitable as benchmarks in this analysis.

The majority of these EECs were set from the highest mean at a single location (N = five replicates at a single location. The smallest NOEC was used in every case except for Cry1Ab determined for foliar-dwelling arthropods (16.7 µg Cry1Ab/g diet [*Chrysoperla carnea*]) and eCry3.1Ab for soil-dwelling invertebrates (10.3 µg eCry3.1Ab/g diet [*Eisenia fetida*]). No adverse effects were observed at the single test concentration; however, in both cases the NOECs were lower than the intended test concentration. This was due to limitations in administering protein in the test system or technical difficulties extracting the protein from a soil-diet matrix. Therefore, NOECs where the intended test concentrations were confirmed, Cry1Ab (200 µg/g diet [Li et al. 2014) and eCry3.1Ab (400 µg/g diet [Raybould et al. 2007]), were used.

The no observed effect level (NOEL) and the daily dietary dose (DDD) related to the exposure scenario in which maize kernels might be consumed by wild mammals or birds were converted from units of µg/g body weight to µg/g fresh weight (FW) to match those of the measured concentrations from the comparative protein expression studies. The derived EECs and NOECs used in this work were converted based on the food intake rate by body weight ratio (0.35) used for cereal seed-eating birds consuming fresh food estimated by Crocker et al. (2002) and the corresponding DDD or NOEL in units of µg/g body weight. The NOELs and DDDs for cereal seed-eating mammals were not converted because those NOELs were higher than those for birds and only the smallest NOEC in every case was of interest.

**Table:** 

Derivation of dietary dose to a concentration	Example derivations (mCry3A)
*NOEC*	*NOEC*
$NOEC = NOEL \div \frac{Food\,intake\,rate}{{bw}}$	$1863\,\upmu {\text{g/g}}\,FW = 652\,\upmu {\text{g/g}}\, bw \div 0.35$
*EEC*	*EEC*
$EEC = DDD \div \frac{Food\,intake\,rate}{{bw}}$	$1.54\,\upmu {\text{g/g}}\,FW = 0.54\,\upmu {\text{g/g}}\, bw \div 0.35$

Each tissue-specific mean protein concentration in each stack and component single event were plotted against each other, relative to a line of identity similar to that presented by Gampala et al. (2017). The line of identity is a y = x line representing the expectation that insecticidal protein concentrations in a single event and associated stacks should equidistantly intersect. The protein and tissue-specific expression data were plotted to scales of linear, log_10,_ compressed linear or log_10,_ or a combination of these, to display both the mean concentrations of stacks and single events with the corresponding NOEC.

## Results

**Fig. 1:**
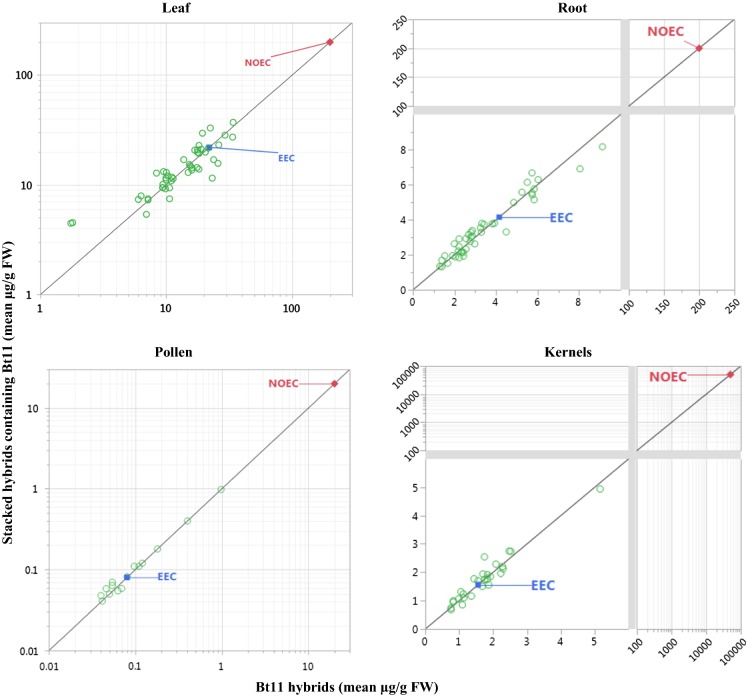
Mean Cry1Ab concentrations in leaves, roots, pollen, and kernels of multiple conventionally-bred maize stacks by those of single event Bt11 maize. Mean Cry1Ab concentrations on a fresh weight (FW) basis are shown in relation to both the estimated environmental concentrations (EEC) set for Cry1Ab in Bt11 maize leaves, roots, pollen, and kernels, and corresponding no observed effect concentrations (NOEC). The EEC is the maximum mean of Cry1Ab concentration (Leaves = 22.02 µg/g FW; Roots = 4.15 µg/g FW; Pollen = 0.08 µg/g FW; Kernels = 1.56 µg/g FW) in Bt11 maize at a particular growth stage and location (Privalle 2003; unpublished [cited in US EPA 2010]). Only the lowest NOEC (foliar non-target arthropods = 200 µg/g FW; soil-dwelling invertebrates = 200 µg/g FW; pollinators = 20 µg/g FW; wild mammals = 50,000 µg/g FW) from effects tests among pertinent species was included (US EPA 2010; Li et al. 2014). Data were plotted to scales of linear, log_10,_ compressed linear or log_10_, or a combination of these, to display both the mean concentrations of stacks and single events with the corresponding NOEC

**Fig. 2:**
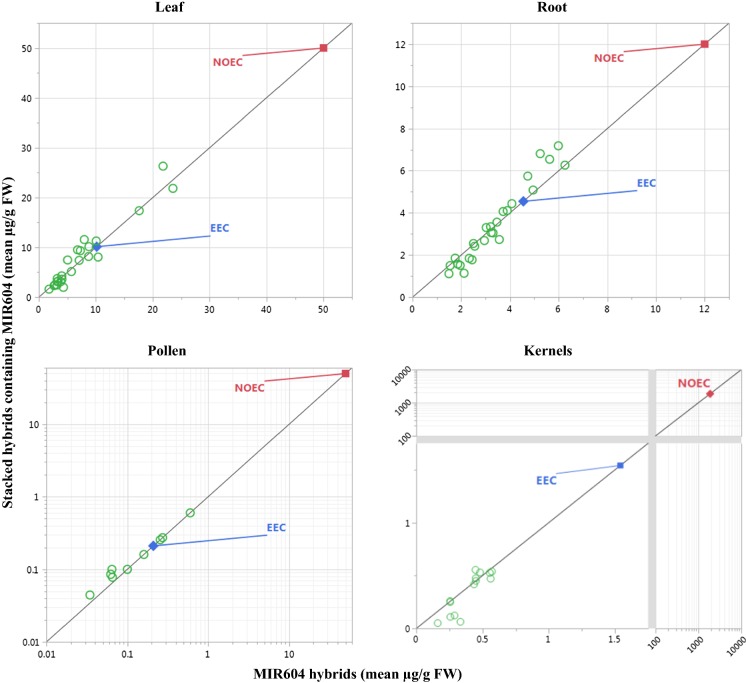
Mean mCry3A concentrations in leaves, roots, pollen, and kernels of multiple conventionally-bred maize stacks by those of single event MIR604 maize. Mean mCry3A concentrations on a FW basis are shown in relation to the estimated environmental concentrations (EEC) set for mCry3A in MIR604 maize and corresponding no observed effect concentrations (NOEC). The EEC is the maximum mean of mCry3A concentration (Leaves = 10.14 µg/g FW; Roots = 4.55 µg/g FW; Pollen = 0.21 µg/g FW; Kernels = 1.54 µg/g FW) in MIR604 maize at a particular growth stage and location (Raybould et al. 2007). Only the lowest NOEC (foliar non-target arthropods = 50 µg/g FW; soil-dwelling invertebrates = 12 µg/g FW; pollinators = 50 µg/g FW; wild mammals = 1863 µg/g FW) from effects tests amongst pertinent species was included (Raybould et al. 2007). Data were plotted to scales of linear, log_10_, or a combination of linear and compressed log_10_ to display both the mean concentrations of stacks and single events with the corresponding NOEC

**Fig. 3:**
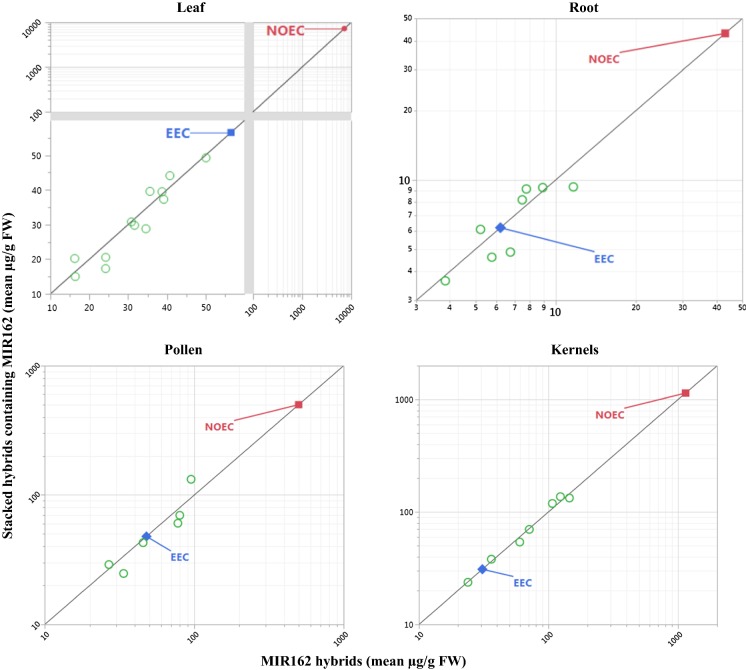
Mean Vip3Aa20 concentrations in leaves, roots, pollen, and kernels of multiple conventionally-bred maize stacks by those of singly event MIR162 maize. Mean Vip3Aa20 concentrations on a FW basis are shown in relation to the estimated environmental concentration (EEC) set for Vip3Aa20 in MIR162 maize and corresponding no observed effect concentration (NOEC). The EEC is the maximum mean of Vip3Aa20 concentration (56.56 µg/g FW; Roots = 6.2 µg/g FW; Pollen = 47.85 µg/g FW; Kernels = 30.9 µg/g FW) in MIR162 maize at a particular growth stage and location (Raybould and Vlachos 2011). Only the lowest NOEC (foliar non-target arthropods = 7250 µg/g FW; soil-dwelling invertebrates = 43.1 µg/g FW; pollinators = 500 µg/g FW; wild mammals = 1143 µg/g FW) from effects tests amongst pertinent species was included (Raybould and Vlachos 2011). Data were plotted to scales of log_10_, and a combination of linear and compressed log_10_ to display both the mean concentrations of stacks and single events with the corresponding NOEC

**Fig. 4:**
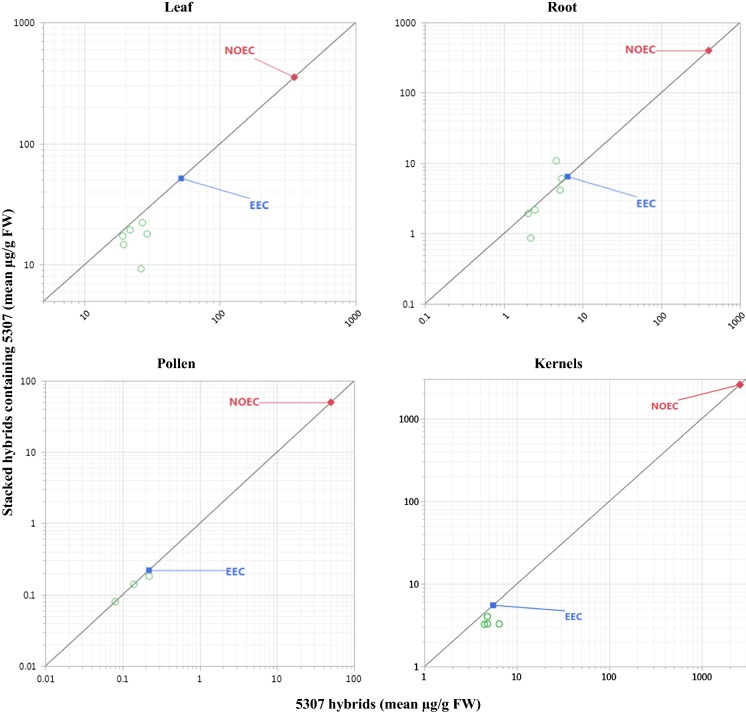
Mean eCry3.1Ab concentrations in leaves, roots, pollen, and kernels of multiple conventionally-bred maize stacks by those of single event 5307 maize. Mean eCry3.1Ab concentrations on a FW basis are shown in relation to the estimated environmental concentration (EEC) set for eCry3.1Ab in 5307 maize and corresponding no observed effect concentration (NOEC). The EEC is the maximum mean of eCry3.1Ab concentration (51.74 µg/g FW; Roots = 6.48 µg/g FW; Pollen = 0.22 µg/g FW; Kernels = 5.53 µg/g FW) in 5307 maize at a particular growth stage and location (Burns and Raybould 2014). Only the lowest NOEC (foliar non-target arthropods = 353 µg/g FW; soil-dwelling invertebrates = 400 µg/g FW; pollinators = 50 µg/g FW; wild mammals = 2571 µg/g FW) from effects tests among pertinent species was included (Burns and Raybould 2014). Data were plotted to log_10_ scale to display the mean concentrations of stacks and single events with the corresponding NOEC

Several mean concentrations for each protein were higher than the EEC used for risk assessment of the corresponding component single event despite (in most cases) the lack of significant difference between stack and single event (Figs. 1, 2, 3, 4). Mean concentrations in samples of a breeding stack were greater than established protein and tissue-type-related EECs for 70 out of 204 comparisons. However, the mean concentration in the plant tissue sample of the corresponding single event was also greater than the corresponding EEC in 65 out of these 70 cases. Most importantly, mean insecticidal protein concentrations in all cases were lower (in most cases several folds lower) than the lowest NOEC of each class of non-target organisms. This means that the MoEs are not eroded to unacceptable levels in the stack risk assessments.

## Discussion

The conclusion of the studies examined collectively in this paper is that the transgenic protein concentrations were generally similar between the stack and the corresponding component single event, indicating a lack of interaction that increases the production of the transgenic insecticidal proteins due to combination of the traits by conventional breeding. These collective statistical comparisons further confirms for each of the proteins analyzed that the expressed insecticidal protein concentrations are not increased by the expression of another when stacked by conventional breeding.

Mean protein concentrations were higher than corresponding EECs in several cases (Figs. 1, 2, 3, 4). In most of those cases, both means for the stack and for the corresponding single event were higher than the previously set EEC. This indicates that the increase was most likely not due to stacking and more likely due to differences in random influencing factors, such as differences in genetic backgrounds and differences in environmental growing conditions. Although many mean concentrations of insecticidal proteins from the stacks in these comparative protein expression studies were higher than EECs used for risk assessment of the single events, the majority were lower. Only one mean was higher than a corresponding EEC by close to fourfold, and none of the rest were higher by more than twofold.

Out of 204 comparisons, none resulted in increased EEC that eroded MoE to unacceptable levels. This demonstrates that robust methods are used for estimating the EECs and for setting test concentrations in NTO effects tests to support the risk assessment of single events. These results provide confidence that the conservative margin between the EEC and test concentrations in NTO effects tests is more than adequate to account for potential increases in transgenic protein expression due to differences in plant genetics or environmental factors. Additionally, this conclusion can be extended to breeding stacks. In other words, this paper demonstrates that transgene stacking by crossing one plant containing a transgene with another plant contain one or more other transgenes is no more likely to lead to a hazardous increase in transgenic protein concentration than is crossing to a nontransgenic variety.

### Considerations to modify testing requirements for breeding stacks

When GM trait stacking by conventional breeding first began, the regulatory community was uncertain about potential impacts on expression for insecticidal proteins. Now we have many experimental studies that indicate a lack of an increase in protein production due to stacking for the Cry1Ab, mCry3A, Vip3Aa20, and eCry3.1Ab proteins by conventional breeding. Therefore, additional comparative expression studies for a new stack with any combination of Bt11, MIR604, MIR162, 5307, and GA21 is no longer necessary to justify transporting existing ERAs for single events to associated stacks. These results may also be useful to consider during problem formulation for combination of transgenes encoding similar insecticidal proteins.

If concern exists through problem formulation that production of transgenic proteins may increase due to interactions with other transgenic proteins combined through conventional breeding, testing could address uncertainty. However, the uncertainty should be related to a plausible possibility to erode a margin of exposure to unacceptable levels due to increased transgenic protein expression. For example, instead of a comparative protein expression study, one might simply measure the insecticidal protein concentration in relevant tissue types of a breeding stack and compare the exposure derivative to relevant existing NOECs.

## Conclusion

The results of the expression studies reported herein conducted to measure and compare transgenic protein concentrations in tissues of the various stacked maize hybrids to those in corresponding component single event maize hybrids summarized herein support the hypothesis that transgenic protein concentrations do not increase to biologically relevant levels due to stacking by conventional breeding. The multitude of tests that corroborate this hypothesis indicate that further tests would not provide new information to inform risk assessment on stacks with any combination of the events Bt11, MIR604, MIR162, or 5307. The collective results from these expression studies also reaffirms that the EECs and test concentrations in NTO effects tests set for risk assessment of single-event crops were robust, confirmed by comparison to the corresponding measured protein concentrations that incorporate variability introduced by different environmental growing conditions and genetic backgrounds across studies. That variability is well contained by setting test concentrations in NTO effects tests at appropriate levels such that margins of exposure are not eroded to unacceptable levels in stack risk assessments. These learnings support that ERAs for single events are transportable to associated breeding stacks without the need for comparative protein expression testing.

## Footnotes

1 Because this paper describes work performed and evaluated by Syngenta scientists, data on the insecticidal proteins Cry1F, Cry34, and Cry35 generated from analysis of five distinct stacks containing at least one of the Corteva Agrosciences products, TC1507 and DAS-59122-7 were not included.

2 0.1 M sodium tetraborate decahydrate, 0.2% Polyvinylpyrrolidine, 7.69 mM sodium azide, 1.2% concentrated hydrochloric acid, 0.5% Tween 20, Complete Protease Inhibitor Cocktail Tablet (added on day of extraction).
